# Multifaceted Effects of Thymoquinone on Platelet Calcium Homeostasis

**DOI:** 10.3390/cells14221827

**Published:** 2025-11-20

**Authors:** Natalia Rukoyatkina, Igor Mindukshev, Diana M. Mikhailova, Mikhail A. Panteleev, Stepan Gambaryan

**Affiliations:** 1 Sechenov Institute of Evolutionary Physiology and Biochemistry, Russian Academy of Sciences, 44 Thorez Ave., 194223 Saint Petersburg, Russia; natalia.rukoyatkina@gmail.com (N.R.); iv_mindukshev@mail.ru (I.M.); mikhailowa.dm@gmail.com (D.M.M.); 2 Center for Theoretical Problems of Physico-Chemical Pharmacology, Russian Academy of Sciences, 30 Srednyaya Kalitnikovskaya St., 109029 Moscow, Russia; mapanteleev@yandex.ru

**Keywords:** platelets, calcium, aggregation, thymoquinone

## Abstract

**Highlights:**

**What are the main findings?**

- Thymoquinone (TQ) strongly and acutely inhibited agonist-induced platelet activation and aggregation. TQ differentially regulates GPCR and GPVI receptor activation by enhancing [Ca^2+^]_i_ mobilization by GPCR-induced activation and inhibition of GPVI-induced [Ca^2+^]_i_ mobi-lization.
- Presented data are the first example in which complete inhibition of ADP- and Trap-6-, but not CRP-induced, aggregation is accompanied by high [Ca^2+^]_i_ levels.

**What is the implication of the main finding?**

- TQ could be a valuable molecule for the analysis of calcium homeostasis in platelets and other cells.
- TQ is now considered a promising therapeutic agent against cancer, and our results, that TQ is a potent inhibitor of platelets, should be taken into account, especially in pathological situations with possible bleeding complications.

**Abstract:**

Thymoquinone (TQ), the main bioactive ingredient of *Nigella sativa*, exhibits numerous pharmacological activities and is used for the prevention of many diseases including hypertension and cancer. However, information concerning the effects of TQ on platelets is limited. In this study, we used the upgraded laser microparticle analyzer LaSca-TMF for simultaneous analysis of platelet shape change, aggregation, and changes in [Ca^2+^]_i_. We showed that TQ acutely inhibited platelet aggregation induced by ADP, Trap-6, and CRP; however, the rise of [Ca^2+^]_i_ was inhibited only in CRP-stimulated platelets, but not in ADP- or Trap-6-stimulated ones. DTT, a thiol-reducing agent, prevented TQ-induced effects in platelets, indicating that protein disulfide isomerases could be involved in the regulation of TQ effects on platelets. Our results, for the first time, demonstrated acute inhibitory effects of TQ on platelet activation induced by GPCRs and ITAM-containing receptors, which were independent of PKA and caspase-3 activation. To the best of our knowledge, this is the first example in which complete inhibition of ADP- and Trap-6-, but not CRP-induced, aggregation is accompanied by high [Ca^2+^]_i_ levels. Additional experimental approaches are required to explain some effects of TQ on calcium homeostasis and TQ could be a valuable molecule for the analysis of calcium homeostasis in platelets and other cells.

## 1. Introduction

Thymoquinone (TQ) as the main bioactive ingredient of *Nigella sativa* exhibits numerous pharmacological activities through regulation of diverse intracellular signaling mechanisms [1]. In several studies on different cell types, it was shown that TQ inhibited the PI3K/PKB pathway [2,3,4], activated AMPK, and inhibited mTOR and S6K [5,6,7,8,9]. Regarding MAP kinases, TQ, depending on the cell type, could inhibit or activate ERK1/2 and p38 MAP kinases [10,11,12,13,14,15]. Through involvement of MAP kinases, NF-kB [16,17], JAK/STAT [18,19] pathways, and other transcription factors, TQ can regulate transcription of many mRNAs. Accordingly, TQ has numerous pharmacological properties, including antioxidant, anti-inflammatory, antihypertensive, antidiabetic, anti-hepatotoxic, hypoglycemic, and lipid-lowering [20]. TQ is used for the treatment and prevention of many diseases, including infections, amenorrhea, dyspepsia, diarrhea, fever, dizziness, hypertension, and cancer. Numerous studies in different cancer cell lines and animal models have described beneficial effects of TQ on cancer development and metastasis formation [21,22,23].

Platelets, in addition to their traditional role in hemostasis, are now recognized as important regulators of inflammation, atherosclerosis, and cancer [24,25,26]. TQ is involved in the regulation of all the aforementioned processes; therefore, we expected that this substance could regulate platelet function. However, information concerning the effects of TQ on platelets is limited. Previously, we [27] and others [28] showed that long-term (30–60 min) incubation of platelets with TQ induces apoptosis through activation of caspase-3. Many anticancer drugs, especially those that induce apoptosis of cancer cells, lead to the development of thrombocytopenia [29,30] whereas, there are no indications in the literature that TQ induces thrombocytopenia, except for one case report [31] without an analysis of the mechanisms. TQ-induced cell death (apoptosis, necroptosis) in different cancer cell lines [32,33,34,35] and platelets [28] is accompanied by elevated intracellular calcium concentration [Ca^2+^]_i_; however, the molecular mechanisms of calcium elevation are not fully defined. In DLBCL cell lines, TQ-induced [Ca^2+^]_i_ elevation is mediated by depletion of the endoplasmic reticulum (ER) and activation of store-operated calcium entry (SOCE) [32]. In platelets, [Ca^2+^]_i_ is regulated by two opposite mechanisms: those responsible for the elevation of [Ca^2+^]_i_ from intracellular stores (dense tubular system (the equivalent of the ER), acidic endo-lysosomes, and mitochondria), that, in turn, activate SOCE and transient receptor potential channels (TRPCs), and those that are responsible for the lowering of [Ca^2+^]_i_ concentration, including sarcoplasmic/endoplasmic Ca^2+^ ATPases (SERCA) and the mitochondrial calcium uniporter (MCU) [36], that pump Ca^2+^ back into intracellular stores, and plasma membrane Ca^2+^ ATPases (PMCAs), that extrude Ca^2+^ out of platelets [37,38]. Mitochondrial calcium overloading and the opening of mitochondrial permeability transition pores activate processes leading to cell death [39,40].

In this study, we analyzed TQ-induced calcium signaling in platelets and showed that elevation of [Ca^2+^]_i_ is independent of extracellular calcium. Despite the elevation of [Ca^2+^]_i_, TQ acutely inhibited platelet aggregation, integrin αIIbβ3 activation, and P-selectin surface expression independently of PKA activation. We also showed that TQ differentially regulated GPCR- and ITAM-induced calcium mobilization.

## 2. Materials and Methods

### 2.1. Ethics Approval

The research was conducted according to the Declaration of Helsinki and approved by the Ethical Committee of the Sechenov Institute of Evolutionary Physiology and Biochemistry of the Russian Academy of Sciences. Human blood was obtained from healthy donors by venipuncture after signing written informed consent (protocol no. 03–02 from 28 February 2024).

### 2.2. Materials

TQ, Calcein-AM (C-AM), iloprost, thapsigargin, THBQ (tetrahydroxy-1,4-benzoquinone), ADP, dithiothreitol (DTT), TRAP-6, and working buffer components (HEPES, NaCl, KCl, MgCl_2_, D-glucose, EGTA, CaCl_2_) were purchased from Sigma-Aldrich (Darmstadt, Germany). The chemical structures of TQ, thapsigargin, THBQ, and DTT are presented in Supplementary Figure S1. Fluo-3-AM was from Invitrogen (Carlsbad, CA, USA); fibrinogen Alexa Fluor 647 and DCF-DA were from Molecular Probes (Göttingen, Germany); PE-conjugated CD62P, CD41, and Annexin-V were from BD Bioscience (Heidelberg, Germany); the cysteine-containing, cross-linked collagen-related peptide (CRP) was kindly provided by Prof. R.W. Farndale (University of Cambridge, Cambridge, UK).

All used stock solutions and their final concentrations were prepared as presented in the Table 1.

### 2.3. Preparation of Platelet-Rich Plasma (PRP) and Washed Human Platelets (WPs)

Human platelets were obtained from venous blood collected by caudal venipuncture in Citrate 9NC (0.106 mol/L/3.2%) S-monovette tubes (Sarstedt, Nümbrecht, Germany) with the addition of 1 mM EGTA and centrifuged at 300× *g* (centrifuge ELMI-50CM, Elmi, Riga, Latvia) for 8 min at RT. Supernatant (PRP) was diluted in the HEPES buffer (150 mM NaCl, 3 mM KCl, 1 mM MgCl_2_, 5 mM D-glucose, 10 mM HEPES, pH 7.4), and used for aggregation and [Ca^2+^]_i_ analysis. For the preparation of WPs, PRP was centrifuged at 2400 rpm for 4 min at RT, washed once in CGS buffer (120 mM NaCl, 12.9 mM trisodium citrate, 10 mM D-glucose, pH 6.5), resuspended in the HEPES buffer, and rested for 20 min at RT until the experiment. To monitor the platelet count and parameters, the Medonic-M20 hematological counter (Boule Medical A.B., Stockholm, Sweden) was used.

### 2.4. Analysis of TQ-Induced Aggregation and Shape Change Reaction by the Laser Diffraction Method

TQ-induced platelet activation and aggregation were analyzed by the laser diffraction method (laser microparticle analyzer LaSca-TM, BioMedSystems Ltd., Saint Petersburg, Russia) described in detail in [41,42,43]. Briefly, the laser beam (650 nm) passed through platelets resuspended in HEPES buffer with 2 mM Ca^2+^ (2 × 10^7^ cells/mL final concentration) in the cuvette with continuous stirring (1200 rpm). The original laser diffraction particle analyzer constantly registers the buffer absorbance (at 0 degrees) and the differences after the addition of TQ (40 µM) were less than 1%.

The platelet shape change was characterized by an increase in the light scatter intensity (LSI) at the scattering angle of 1°. Platelet aggregation was characterized by the LSI increase at the scattering angle of 1° with a simultaneous LSI decrease at the scattering angle of 12°. The area under the curve (AUC) during three minutes of reaction was used for calculating the aggregation reaction, and the velocity of platelet shape change (V_shape_) was assessed upon initiation of a shape change (for details, see Supplementary Figure S2A,B).

### 2.5. Analysis of Platelet Ca^2+^ Mobilization

To analyze TQ-induced changes in [Ca^2+^]_i_, the upgraded laser microparticle analyzer LaSca-TMF equipped with a 488 nm laser and FL1 fluorescence detector (527 nm) (BioMedSystems Ltd., Saint Petersburg, Russia), which allows simultaneous detection of [Ca^2+^]_i_, shape change, and aggregation, was used. The method is described in detail in [43]. PRP was incubated with Fluo-3-AM (10 µM, 60 min, RT) in the dark, and then platelets were diluted in HEPES buffer (2 × 10^7^ cells/mL final concentration). Intracellular Fluo-3 was excited at 488 nm, and the emission was registered at 527 nm (FL1). The area under the curve (AUC_Ca_) was calculated to characterize TQ-induced [Ca^2+^]_i_ changes in platelets (for details, see Supplementary Figure S2C). [Ca^2+^]_i_ in nM was calculated according to Supplementary Figure S3A,B. All data from the laser diffraction method were analyzed using the original software LaSca_32 v.1498 (BioMedSystems Ltd., Saint Petersburg, Russia) of the laser particle analyzer LaSca-TMF.

### 2.6. Flow Cytometry Analysis

TQ-induced changes in platelet reactivity (15,000 events) were analyzed by flow cytometry using the CytoFLEX flow cytometer (Beckman Coulter, Brea, CA, USA). Platelets were gated according to CD41-positive events. αIIbβ3 integrin activation was analyzed by fibrinogen Alexa Fluor 647 binding. Fibrinogen (final concentration 15 μg/mL) was added to washed platelets (2 × 10^7^ cells/mL, for 10 min); then TQ (40 μM) was added for different time points, and platelets were stimulated by Trap-6 (10 µM, 2 min). After the indicated time, platelets were fixed by 1% formalin, then washed with PBS, diluted with PBS (1:40), and the median fluorescence intensity (MFI) was registered at the FL6 channel. P-Selectin surface expression was characterized by (PE)-conjugated CD62P antibodies. Antibodies were added to washed platelets (2 × 10^7^ cells/mL, 10 min); then TQ (40 μM) was added for different time points, and platelets were stimulated by thrombin (0.05 U/mL, 2 min). After the indicated time, platelets were fixed by 1% formalin, then washed with PBS, diluted with PBS (1:40), and the MFI was registered at the FL2 channel. Platelet viability was analyzed by a marker of cell esterase activity (C-AM). Platelets (2 × 10^7^ cells/mL final concentration) were incubated with TQ (40 μM, for the indicated time, at 37 °C) and then C-AM (0.2 μM, 10 min) was added to cells. The reaction was stopped by PBS (1:40), and calcein MFI was registered at the FL1 channel. Phosphatidylserine exposure (PS) was measured by Annexin V-PE binding. Platelets were incubated with Annexin-V-PE (1:10) and then TQ (40 μM, for indicated time) was added to the cells. Formation of reactive oxygen species (ROS) in platelets was analyzed by DCF-DA. Washed platelets (2 × 10^7^ cell/mL) were incubated with DCF-DA (10 μM, 30 min) then TQ (40 μM, for indicated time) was added to platelets. Data were analyzed using the original software CytExpert v2.4 (Beckman Coulter, Brea, CA, USA).

### 2.7. Western Blot Analysis

Western blots were performed as described previously [44]. Washed platelets (3 × 10^8^ platelets/mL) were treated with TQ for the indicated time and then lysed with Laemmli sample buffer. Proteins were separated by SDS-PAGE, transferred to nitrocellulose membranes. The membranes were incubated with phospho-Vasodilator-Stimulated Phosphoprotein (VASP) S239 (Clone 16c2) (Nano Tools, Teningen, Germany) antibodies and anti-Actin (# 4970) antibodies (Cell Signaling, Frankfurt, Germany) primary antibodies overnight at 4°C. For visualization of the proteins, goat anti-rabbit or anti-mouse IgG conjugated with horseradish peroxidase were used as secondary antibodies, followed by ECL detection (GE Healthcare, Chicago, IL, USA). Blots were analyzed densitometrically using NIH ImageJ (1.54g) software.

### 2.8. Data Analysis

Statistical analysis was performed in GraphPad Prism v.9 (GraphPad Software Inc., San Diego, CA, USA). The data sets were tested for normality using the Kolmogorov–Smirnov normality test. The differences between the two groups were compared either using Student’s *t*-test or the Mann–Whitney U test. For multiple comparisons, either one-way ANOVA followed by Dunnett’s post hoc test or Kruskal–Wallis test was used. Data are presented as means ± SD. All experiments were performed at least four times (*n* = 4); *p* < 0.05 was considered statistically significant.

## 3. Results

### 3.1. TQ Inhibited Agonist-Induced Platelet Activation

**Figure 1:**
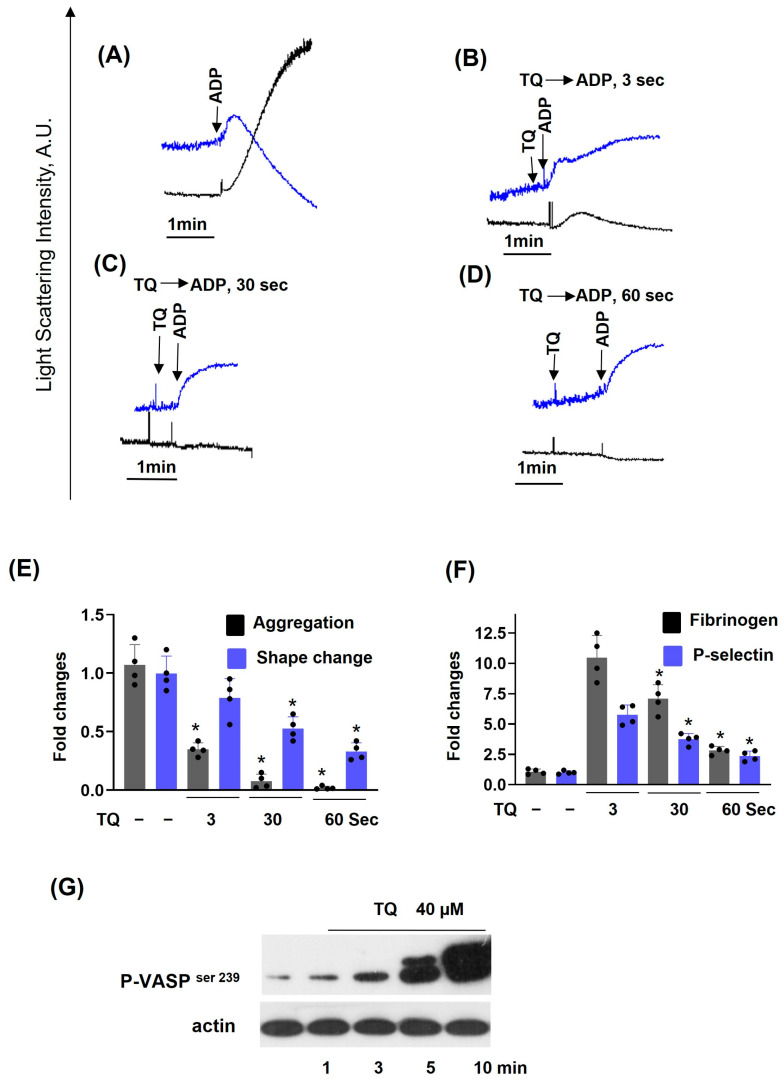
Inhibition of ADP-induced platelet activation is independent of PKA activation. PRP was diluted in HEPES buffer to maintain a platelet concentration of 2 × 10^7^/mL and was stimulated with ADP (1 µM) or preincubated with TQ (40 µM) for the indicated time. Aggregation and shape change reaction were analyzed by LaSca-TM device. Aggregation was analyzed as AUC; shape change was assessed as changes in velocity (V_shape_). LSI AU—light scattering intensity in arbitrary units at 1° for shape change reaction (blue lines) and at 12° for aggregation (black lines). For details, see Supplementary Figure S2A,B. (**A**–**D**) are original records; (**E**,**F**) are the summary data from four independent experiments (four different blood donors). Data (means ± SD, *n* = 4, * significant differences from ADP-alone samples, *p* < 0.05) are presented as fold changes where ADP was taken as 1. Flow cytometry analysis of integrin αIIbβ3 activation and P-selectin expression in washed platelets (WP) stimulated with Trap-6 (10 µM, 2 min) or preincubated with TQ for the indicated time. Data (means ± SD, *n* = 4, * significant differences from Trap-6-alone samples, *p* < 0.05) are presented as fold changes where Trap-6 was taken as 1. (**G**), Western blot analysis of time-dependent TQ-induced VASP phosphorylation in washed platelets. Representative blots from three independent experiments.

**Figure 2:**
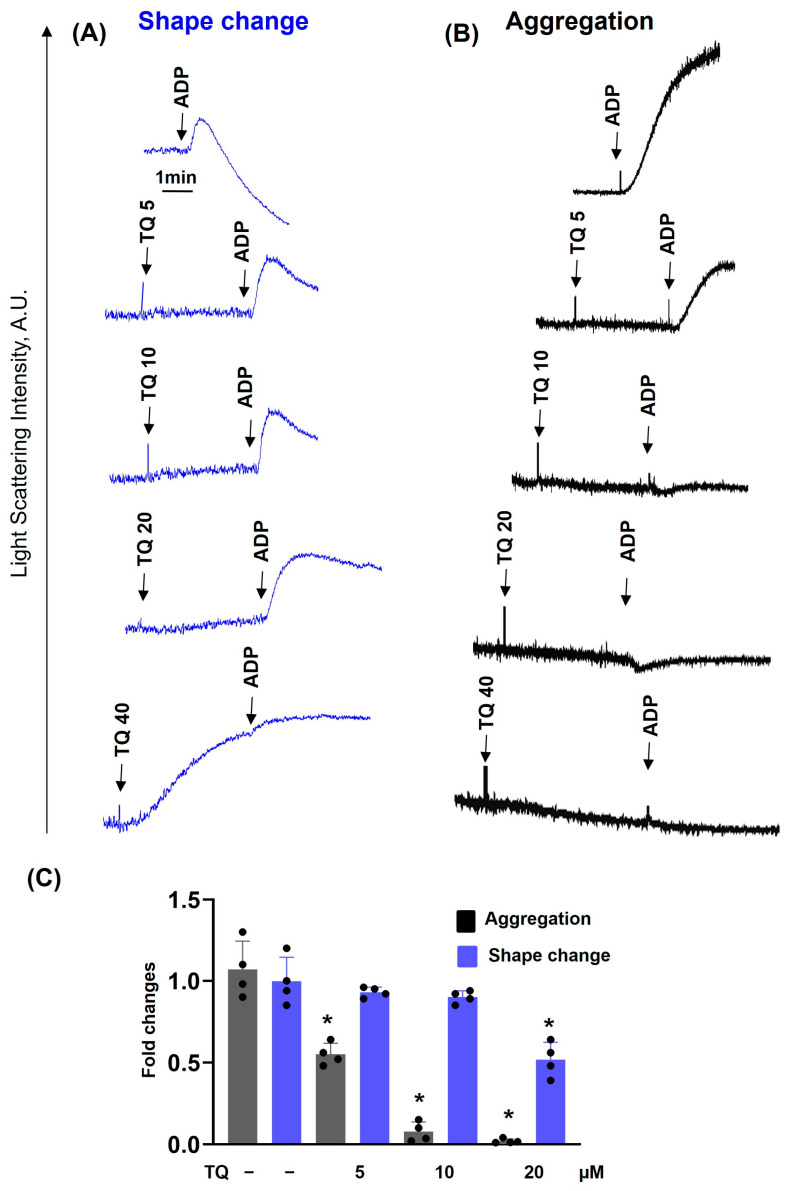
TQ concentration-dependently inhibits ADP-induced platelet aggregation. PRP from the same conditions as in Figure 1A–D was analyzed for aggregation (**A**) and shape change reaction (**B**) in LaSca-TM device. PRP was stimulated with ADP (1 µM) or preincubated with the indicated concentrations of TQ. Shown are original traces from four independent experiments. (**C**) are the summary data from four independent experiments (four different blood donors). Data (means ± SD, *n* = 4, *—significant differences from ADP-alone samples, *p* < 0.05) are presented as fold changes where ADP effect was taken as 1.

**Figure 3:**
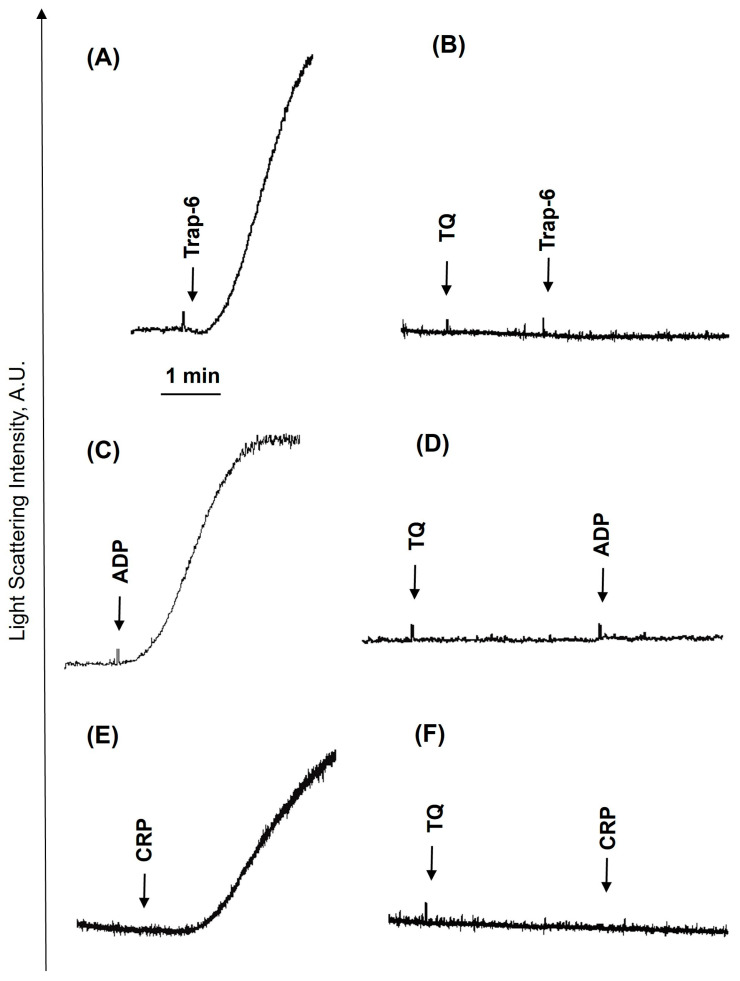
TQ completely inhibits agonist-induced platelet aggregation. PRP from the same conditions as in Figure 1A–D was analyzed for aggregation in LaSca-TM device. Shown are original traces from four independent experiments. PRP was stimulated by 10 µM Trap-6 (**A**), 1 µM ADP (**C**), 10 µg/mL CRP (**E**), or preincubated with TQ (40 µM, 3 min, **B**,**D**,**F**) and then stimulated with agonists.

**Figure 4:**
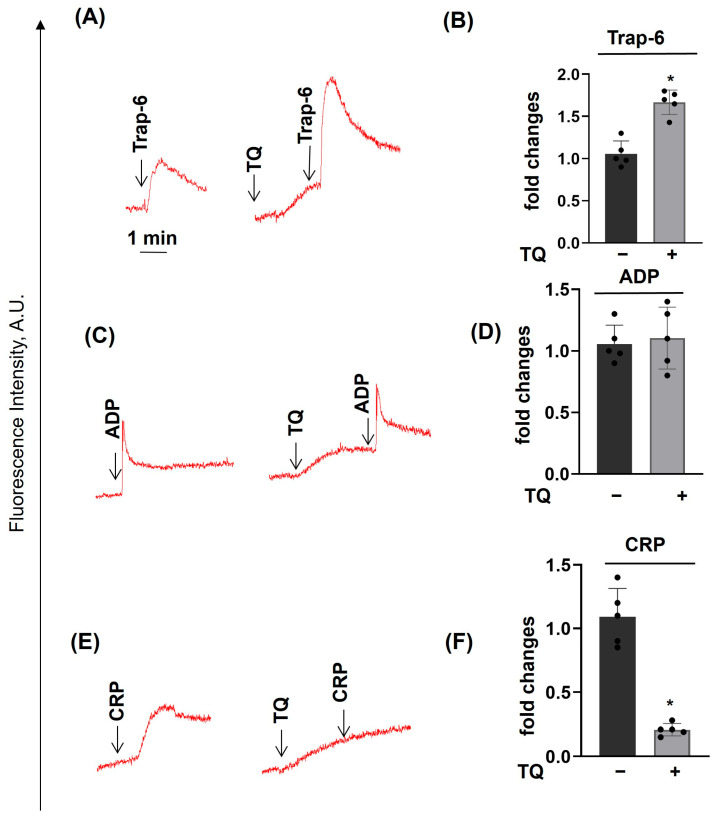
TQ differentially regulates agonist-induced intracellular Ca^2+^ mobilization. PRP was loaded with Fluo-3 AM (10 µM, 40 min), then diluted in HEPES buffer to maintain a platelet concentration of 2 × 10^7^/mL and was stimulated with 10 µM Trap-6 (**A**,**B**), 1 µM ADP (**C**,**D**), 10 µg/mL CRP (**E**,**F**), or preincubated with TQ (40 µM, 3 min), and calcium mobilization was analyzed by LaSca-TMF device (for details, see Supplementary Figure S2C). FI at FL1, AU—fluorescence intensity at FL1 in arbitrary units. TQ significantly enhanced Trap-6-induced [Ca^2+^]_i_ mobilization, had no effect on ADP-induced mobilization, and strongly inhibited CRP-induced platelet calcium mobilization. Left panels are original records; right panels are summary data from four independent experiments (four different blood donors). Data (means ± SD, *n* = 4, *—significant differences from samples without TQ, *p* < 0.05) are presented as fold changes where Trap-6 (**A**), ADP (**C**), and CRP (**E**) were taken as 1.

Previously, it was shown that long-term (30–60 min) incubation with TQ induces platelet apoptosis by activation of caspase-3 [27,28]. We also demonstrated that under these conditions, TQ induces cAMP-independent PKA activation, which corresponds to inhibition of thrombin-induced platelet activation [27]. Here we showed that TQ-induced reduction in platelet viability started only after 30 min of incubation (Supplementary Figure S4). However, whether TQ could acutely inhibit platelet activation induced by different stimuli and whether this inhibition is correlated with PKA activation was not known. First, we analyzed time-dependent inhibition of ADP-induced platelet activation (Figure 1). ADP induced shape change reaction and aggregation (Figure 1A), which were strongly inhibited by simultaneous addition of TQ (Figure 1B) and completely inhibited after 0.5 (Figure 1C) and 1 min (Figure 1D) preincubation with TQ. Inhibition of aggregation (Figure 1E) correlated with inhibition of integrin αIIbβ3 activation and P-selectin surface expression (Figure 1F). Importantly, inhibition of ADP-induced platelet activation was independent of PKA activation because VASP phosphorylation started only after 5 min of incubation with TQ (Figure 1G). TQ concentration-dependently inhibited ADP-induced platelet activation (Figure 2). Even 5 µM of TQ significantly inhibited aggregation, and, starting from 10 µM, aggregation was fully inhibited (Figure 2B,C); shape change reaction was partly inhibited at 20 µM (Figure 2A,C), and 40 µM of TQ induced a shape-change reaction and was not enhanced by stimulation with ADP (Figure 2A). Next, we analyzed whether TQ could inhibit platelet aggregation induced by different agonists. TQ completely inhibited Trap-6 (Figure 3A,B), ADP (Figure 3C,D), and CRP (Figure 3E,F) induced platelet aggregation, indicating that TQ-mediated inhibition of aggregation is independent of the agonists. In all following experiments, 40 µM of TQ was used, because this concentration increases [Ca^2+^]_i_ in platelets (Figure 4).

### 3.2. TQ Differentially Regulates Agonist-Induced Intracellular Calcium Mobilization

**Figure 5:**
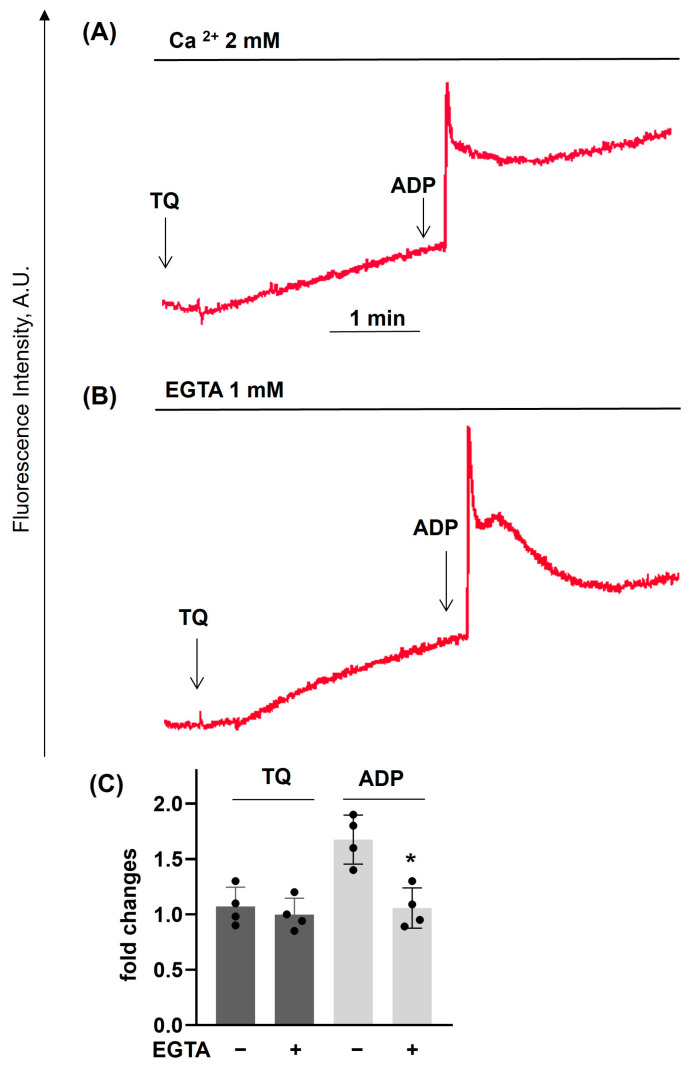
TQ-induced elevation of calcium mobilization is independent of extracellular calcium. PRP prepared as described in Figure 4 was analyzed for calcium mobilization using LaSca-TMF device. (**A**) In the presence of 2 mM calcium in the buffer, (**B**) In the buffer without calcium and 1 mM EGTA. PRP was incubated with TQ (40 µM, 3 min), then ADP (1 µM was added. (**A**,**B**) are the original traces from four independent experiments, (**C**) Is the summary data from these experiments. Data in (**C**) (means ± SD, *n* = 4, *—significant differences from samples in the buffer with calcium) are presented as fold changes where samples in the presence of calcium were taken as 1. TQ-induced elevation of intracellular calcium is not dependent on extracellular calcium, whereas ADP-induced mobilization is significantly reduced without extracellular calcium.

Previously [28], an increase of [Ca^2+^]_i_ in platelets during 30 min of incubation with TQ was demonstrated. However, it was not known whether TQ could acutely increase [Ca^2+^]_i_, whether TQ could modulate agonist-induced [Ca^2+^]_i_ increase, and whether the increase of [Ca^2+^]_i_ is dependent on extracellular calcium concentration. TQ significantly enhanced Trap-6-induced [Ca^2+^]_i_ (Figure 4A,B), had no effect on ADP-induced [Ca^2+^]_i_ (Figure 4C,D), and strongly inhibited the CRP-induced effect (Figure 4E,F). Next, we showed that TQ-induced [Ca^2+^]_i_ increase was independent of extracellular calcium (buffer with EGTA, Figure 5A–C). We also calculated TQ- and ADP-induced [Ca^2+^]_i_ in nM and found that TQ (40 µM) in calcium and EGTA buffer increases [Ca^2+^]_i_ up to 200 ± 36 nM, and ADP (2 µM) in buffer with calcium up to 700 ± 65 nM (Supplementary Figure S3). These data indicate that (i) TQ-induced increase of [Ca^2+^]_i_ is mediated by the release of calcium from intracellular stores (increase of [Ca^2+^]_i_ in the buffer with EGTA); and (ii) regulation of different receptors (P2Y12, P2X1, Par4, GPVI)-induced [Ca^2+^]_i_ by TQ is mediated by its effects on diverse mechanisms that govern intracellular calcium homeostasis.

### 3.3. TQ Potentiated the Effects of Thapsigargin and THBQ

**Figure 6:**
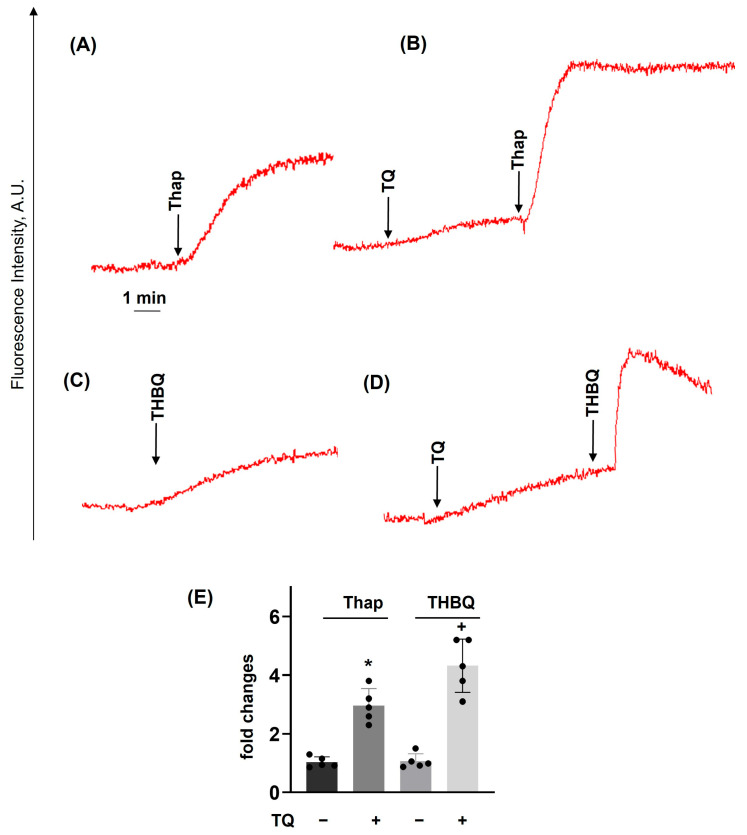
TQ potentiates thapsigargin- and THBQ-induced Ca^2+^ mobilization. PRP prepared as described in Figure 4 was analyzed for calcium mobilization using the LaSca-TMF device. (**A**,**B**), PRP was incubated with thapsigargin (Thap, 10 μM); (**B**), preincubated with TQ (40 μM, 3 min) (**C**,**D**), PRP was preincubated with THBQ (20 μM), preincubated with TQ (40 μM, 3 min). (**A**–**D**) are original traces from five independent experiments. Data on (**E**) (means ± SD, *n* = 5, *—significant differences, *p* < 0.05, from thapsigargin samples; + significant differences, *p* < 0.05, from THBQ samples) are presented as fold changes in which thapsigargin or THBQ alone is taken as 1.

TQ-induced elevation of [Ca^2+^]_i_ was mediated by the release of calcium from intracellular stores (Figure 5). Two isoforms of Ca^2+^ ATPases (SERCA) that pump Ca^2+^ back into intracellular stores are identified in platelets. SERCA2b is associated with the dense tubular system, and SERCA3 is located in acidic endo-lysosomes [45]. Thapsigargin is an established inhibitor of SERCA2b, and THBQ inhibits SERCA3. Inhibition of SERCA2b (Figure 6A) and SERCA3 (Figure 6C) induces elevation of [Ca^2+^]_i_, with a more potent effect for thapsigargin. Preincubation with TQ in both cases (Figure 6B,D) strongly potentiated the increase of [Ca^2+^]_i_, indicating synergism between these two mechanisms in platelet [Ca^2+^]_i_ regulation.

### 3.4. PKA Activation Completely Inhibited Agonist-Induced [Ca^2+^]_i_, Whereas TQ Partly Reversed This Effect

**Figure 7:**
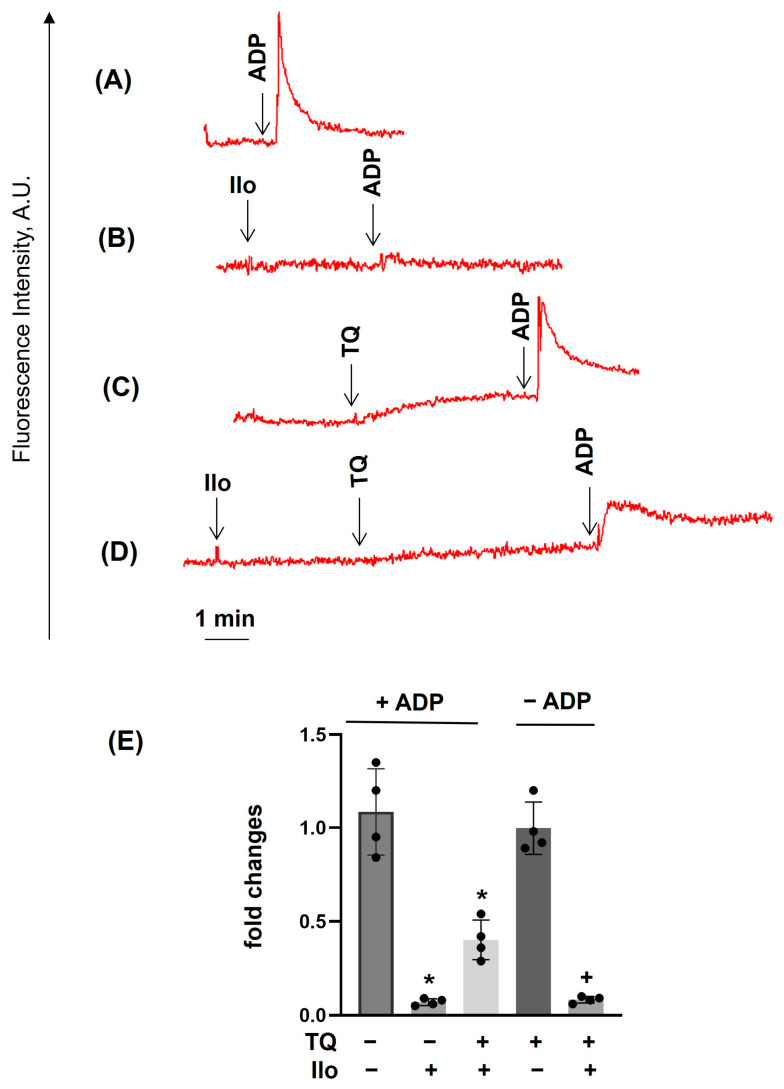
TQ partly reverses the iloprost-mediated inhibition of ADP-induced calcium mobilization. PRP prepared as described in Figure 4 was analyzed for calcium mobilization using the LaSca-TMF device. PRP was stimulated by 1 µM ADP (**A**–**D**); (**B**) preincubated with iloprost (Ilo, 5 nM, 2 min); (**C**) preincubated with TQ (40 µM, 3 min); or in combination of Ilo + TQ, (**D**). (**E**) The summary data from these experiments. (**A**–**D**) are original traces from four independent experiments. Data in (**E**) (means ± SD, *n* = 4, *—significant differences from ADP in +ADP samples; +—significant differences from TQ in −ADP samples, *p* < 0.05) are presented as fold changes in which +ADP, ADP is taken as 1, and in −ADP, TQ-induced elevation of intracellular calcium is taken as 1.

**Figure 8:**
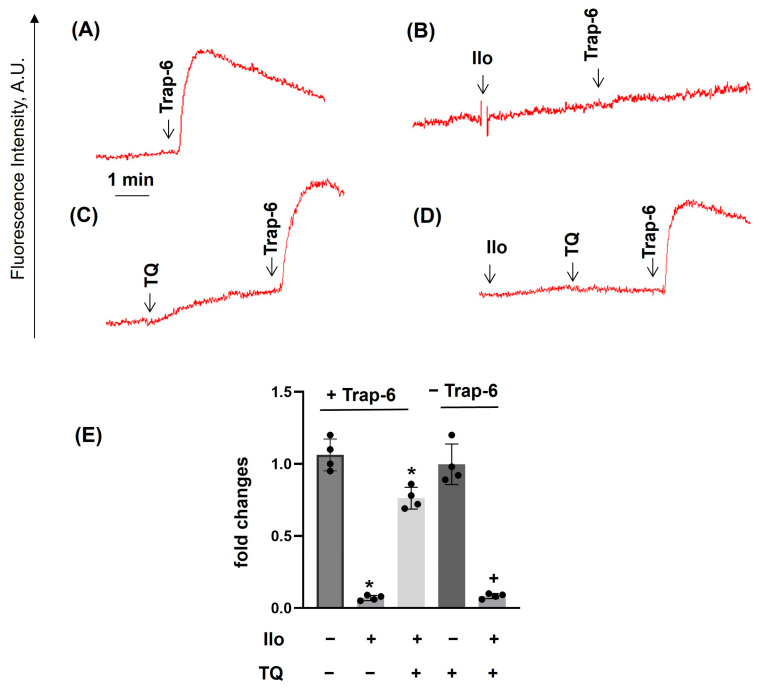
TQ strongly reverses iloprost-mediated inhibition of Trap-6-induced calcium mobilization. PRP prepared as described in Figure 4 was analyzed for calcium mobilization using the LaSca-TMF device. PRP was stimulated by 10 µM Trap-6 (**A**–**D**); (**B**) preincubated with iloprost (Ilo, 5 nM, 2 min); (**C**) preincubated with TQ (40 µM, 3 min); or in combination of Ilo + TQ, (**D**). (**A**–**D**) are original traces from four independent experiments. (**E**) The summary data from these experiments. Data in (**E**) (means ± SD, *n* = 4, *—significant differences from Trap-6 in +Trap-6 samples; +—significant differences from TQ in −Trap-6 samples, *p* < 0.05) are presented as fold changes in which +Trap-6, Trap-6 is taken as 1, and in −Trap-6, TQ-induced elevation of intracellular calcium is taken as 1.

**Figure 9:**
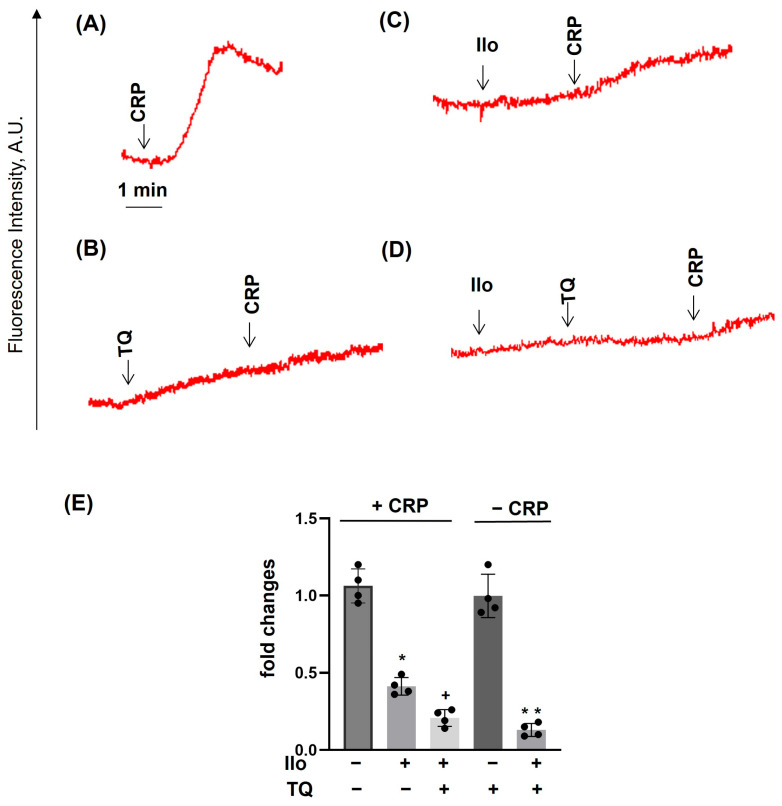
TQ did not reverse the iloprost-mediated inhibition of CRP-induced calcium mobilization. PRP prepared as described in Figure 4 was analyzed for calcium mobilization using the LaSca-TMF device. PRP was stimulated by 10 µg/mL of CRP (**A**–**D**); (**B**), preincubated with iloprost (Ilo, 5 nM, 2 min); (**C**), preincubated with TQ (40 µM, 3 min); or in combination of Ilo + TQ, (**D**). (**A**–**D**) are original traces from four independent experiments. (**E**)—is the summary data from these experiments. Data in (**E**) (means ± SD, *n* = 4, * significant differences from CRP in +CRP samples; +—significant differences from TQ in −CRP samples, *p* < 0.05) are presented as fold changes, in which in +CRP, CRP is taken as 1, and in −CRP, TQ-induced elevation of intracellular calcium is taken as 1.

PKA activation inhibited platelet responses, including [Ca^2+^]_i_ mobilization [46]. Activation of PKA by iloprost completely inhibited ADP- (Figure 7A,B), Trap-6- (Figure 8A,B), and TQ (Figure 7D, Figure 8D and Figure 9D)-induced [Ca^2+^]_i_ mobilization, whereas the CRP-induced effect was not completely inhibited (Figure 9A,B). Interestingly, TQ added after iloprost partly (Figure 7D; in the case of ADP) and more effectively (Figure 8D; in the case of Trap-6) restored [Ca^2+^]_i_ and had no significant effect in the case of CRP (Figure 9). These data confirm that GPCRs and ITAM containing receptors are differentially involved in the regulation of calcium homeostasis in platelets.

### 3.5. Dithiothreitol Prevented TQ-Induced Effects in Platelets

**Figure 10:**
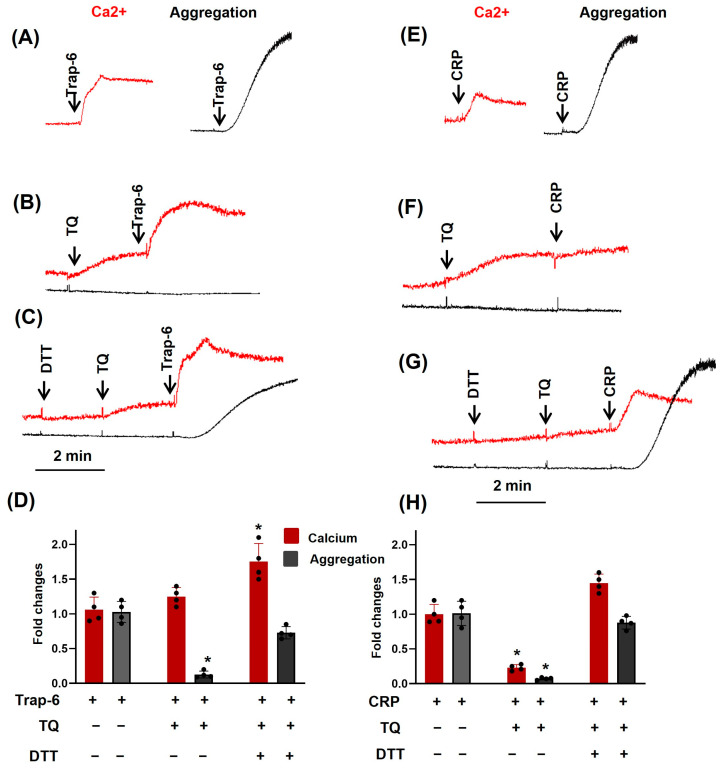
Dithiothreitol (DTT—a thiol-modifying agent) differentially regulates TQ-induced Trap-6 and CRP effects in platelets. PRP prepared as described in Figure 4 was analyzed for calcium mobilization (red) and aggregation (black) using the LaSca-TMF device. PRP was stimulated with 10 µM TRAP-6 (**A**–**D**); preincubated with TQ (40 µM, 3 min) (**B**,**D**); or preincubated with 100 µM DTT, then with TQ (**C**,**D**). PRP was stimulated with 10 µg/mL CRP (**E**–**I**); preincubated with TQ (40 µM, 3 min) (**F**,**I**); or preincubated with 100 µM DTT, then with TQ (**G**,**I**). Platelet aggregation and calcium mobilization were analyzed by the LaSca-TMF device. (**A**–**G**) are original records. Data in (**D**) (means ± SD, *n* = 4) are summary results where Trap-6 is taken as 1, * significant differences from −/+ TQ. Data in (**I**) (means ± SD, *n* = 4) are summary results where CRP is taken as 1, * significant differences from −/+ TQ.

Because TQ inhibitory effects on platelets started immediately (from 3 s after addition; see Figure 1), we proposed that these effects could be connected with modification of the surface proteins. TQ targets could be the family of vascular thiol isomerases (VTIs) that include protein disulfide isomerases (PDIs), endoplasmic reticulum protein 5 (ERp5), ERp46, ERp57, ERp72, and thioredoxin-related transmembrane protein 1 (TMX1). VTIs play an essential role in platelet aggregation and the formation of thrombus [47]. In platelets, several isoforms of VTI are expressed, which are involved in negative as well as positive regulation of platelet activation [47,48]. In several papers, it was shown that some quinones could directly affect the thiol groups in cysteine residues [49,50,51], and we tested whether DTT—a thiol reducer [52] could affect TQ-induced platelet inhibition. DTT itself at a concentration of 100 µM had no direct effect on platelet activation. It had no effect on [Ca^2+^]_i_ mobilization and completely prevented TQ-mediated inhibition of ADP-stimulated aggregation (Supplementary Figure S5). Similarly, it had no effect on [Ca^2+^]_i_ mobilization and partly prevented TQ-mediated inhibition of Trap-6-stimulated aggregation (Figure 10A–D). DTT prevented TQ-induced inhibition of aggregation and calcium mobilization in CRP-stimulated platelets (Figure 10E–I). These data supported the interpretation that TQ effects on cells, at least partly, are mediated by its action on PDIs.

## 4. Discussion

Antithrombotic activity of twenty-one different quinones was recently documented [53,54]. Among them, TQ, the primary bioactive compound of the plant *Nigella sativa*, is now considered a promising therapeutic agent against cancer [55]. However, the molecular mechanisms of TQ in platelets are not clear. Previously, it was demonstrated that TQ induced [Ca^2+^]_i_ mobilization, which leads to activation of caspase-3 and subsequent platelet apoptosis [28]. Increase of [Ca^2+^]_i_ by most agonists is a prerequisite for platelet activation; therefore, we expected that TQ could (probably not strongly) activate platelets. Surprisingly, our first results showed that TQ acutely (from 3 s) strongly inhibited ADP-induced aggregation (Figure 1). Inhibition of platelets could be connected with a reduced viability (monitored by cellular esterase activity, Calcein-AM test), or procoagulant platelets formation and strong PS surface exposure. In our experiments, TQ reduced esterase activity after 30 min of incubation (Supplementary Figure S4) and induced PS exposure starting after 10 min of incubation (Supplementary Figure S6); therefore, these mechanisms could not be connected with the acute inhibitory effect of TQ. TQ is also involved in the generation, as well as scavenging, of reactive oxygen species (ROS) [55], which plays an important role in platelet activation [56] and we tested whether TQ could induce ROS production in platelets and showed that strong ROS generation induced by TQ started after 10 min of incubation (Supplementary Figure S7), therefore this mechanism is also not involved in the acute TQ effect on platelets. Surprisingly, TQ strongly potentiated Trap-6-induced ROS production (Supplementary Figure S8). Additional experimental approaches are needed to explain this unexpected interaction between TQ-induced ROS production, platelet inhibition, and [Ca^2+^]_i_ mobilization.

Regulation of [Ca^2+^]_i_ in platelets is mediated by complex reactions starting from the release of calcium from intracellular stores and activation of SOCE and TRPC. Activation of GPCR (ADP, Trap-6) and ITAM-containing receptors (CRP) differentially regulates the release of calcium from intracellular stores. GPCR (ADP, Trap-6) stimulates platelets by both Gq and Gi signaling mechanisms. Gq, by activation of PLCβ, increases the concentration of inositol-1-4-5 trisphosphate, induces the release of calcium from intracellular stores. Activation of Gi inhibits adenylate cyclase (reduces cAMP concentration) and stimulates calcium release by activation of PI3 kinase. Binding of CRP to GPVI induces activation of Src family kinases, phosphorylation of Syk, which initiates downstream signaling, activation of PLCγ2, and an induced increase in [Ca^2+^]_i_ [57,58]. Interestingly, TQ enhanced Trap-6-induced increase of [Ca^2+^]_i_, did not affect ADP-induced increases, and strongly inhibited CRP-induced increase of [Ca^2+^]_i_ (Figure 4), and at the same time completely inhibited platelet activation induced by all tested agonists. TQ-induced elevation of [Ca^2+^]_i_ occurred in the EGTA buffer, indicating that it is mediated by release from the intracellular stores, and which isoform of PLC is activated by TQ remains to be elucidated. Inhibition of CRP-induced elevation of [Ca^2+^]_i_ could be explained by a direct inhibitory effect on GPVI receptor, and the elevation of Trap-6-induced [Ca^2+^]_i_ might be connected with the additive effect of TQ on PLCγ2 activation. Another possible explanation of TQ effects on GPCR-induced [Ca^2+^]_i_ mobilization could be connected with the fact that TQ, as a lipophilic compound, would preferentially target membrane-associated proteins involved in Ca^2+^ transport. Additional experimental approaches are needed to prove these assumptions.

Another important mechanism involved in platelet calcium homeostasis is connected with Ca^2+^ ATPases that pump calcium out of the cells (PMCAs) and those that pump it back to intracellular stores (SERCA). We tested whether TQ could influence SERCA activity and found that TQ strongly potentiated the effects of inhibition of SERCA2b (thapsigargin) and SERCA3 (THBQ). TQ-induced increase of [Ca^2+^]_i_ was independent of extracellular calcium (Figure 5) and was potentiated by inhibition of SERCA. One of the possible explanations for this could involve inhibition of PMCAs.

PKA activation inhibited agonist-induced calcium mobilization in platelets by modulation of the mechanisms, including release from intracellular stores, transport from the cell membrane, and efflux from the cell, as well as filling back of stores. TQ-induced elevation of [Ca^2+^]_i_ was mediated by its transport from the intracellular stores (not dependent on extracellular calcium) and was also completely inhibited by PKA activation (Figure 7D). In contrast to GPCR activation, platelet activation and [Ca^2+^]_i_ mobilization induced by CRP were not completely inhibited by PKA [59,60], whereas TQ completely inhibited CRP-induced platelet aggregation (Figure 3), strongly reduced [Ca^2+^]_i_ mobilization (Figure 4), and a combination of iloprost with TQ more potently inhibited [Ca^2+^]_i_ mobilization. These data indicate that TQ is a more potent inhibitor of GPVI than cAMP signaling. However, how TQ partly restored ADP-induced and very strongly restored Trap-6-induced calcium mobilization in the presence of iloprost remains a puzzle that is difficult to explain by known mechanisms of calcium homeostasis regulation.

DTT in combination with platelet agonists enhanced aggregation [61] only at very high (mM) concentrations [62]. In our experiments, DTT (100 µM) completely prevented TQ-mediated platelet inhibition and, in the case of CRP, even inhibition of calcium mobilization. These data support previous observations that quinones can directly affect the function of glutathione and VTI [50,51]. Inhibition of platelet aggregation by other quinones (1,4-benzoquinone, 1,4-naphtoquinone) is connected with the depletion of platelet glutathione (GSH) [49]. Whether inhibition of platelet activation by TQ is also mediated by the depletion of GSH remains to be elucidated.

## 5. Conclusions

Our results for the first time demonstrated acute inhibitory effects of TQ on platelet activation induced by GPCRs and ITAM-containing receptors, which were independent of PKA and caspase-3 activation. To the best of our knowledge, this is the first example in which complete inhibition of ADP- and Trap-6- but not CRP-induced, aggregation is accompanied by high [Ca^2+^]_i_ levels. Additional experimental approaches are required to explain some effects of TQ in calcium homeostasis, and TQ could be a valuable molecule for the analysis of calcium homeostasis in platelets and other cells. Because TQ is now considered a promising therapeutic agent against cancer [55], our results, that TQ is a potent inhibitor of platelets, should be taken into account, especially in pathological situations with possible bleeding complications.

## Figures and Tables

**Table 1:** Preparation of Stock Solutions.

Compound	Stock Solutions Solvents	Stock Solutions Concentrations	Final Concentrations
TBHQ	DMSO **	20 mM	20 μM
TQ *	DMSO	40 mM	5–40 μM
Thapsigargin	DMSO	1 mM	1 μM
TRAP-6	DMSO	10 mM	10μM
ADP	Water	10 mM	1 μM
CRP	PBS	5 mg/mL	10 µg/mL
Iloprost	Ethanol	55 μM	5 nM
DTT	Water	10 mM	100 μM

* For each experiment, the TQ solution was freshly prepared and stored in the dark. ** To exclude solvent-related effects on platelets, the equivalent concentrations of DMSO/Ethanol were added to control samples. DMSO/Ethanol did not affect [Ca^2+^]_i_ concentration, shape change, aggregation, and cell viability of platelets.

## Data Availability

The data underlying this article will be shared at a reasonable request to the corresponding author.

## Supplementary Materials

The following supporting information can be downloaded at: https://www.mdpi.com/article/10.3390/cells14221827/s1, Figure S1. Structural formulas of TQ, THBQ, thapsigargin, and DTT; Figure S2. Analysis of platelets aggregation, shape change, and [Ca^2+^]_i_ mobilization by the Laser Diffraction Method; Figure S3. Calculation of [Ca^2+^]_i_ concentration by the Laser Diffraction Method; Figure S4. TQ decreases cell esterase activity, starting from 30 min of incubation; Figure S5. Dithiothreitol reverses TQ-induced inhibition of ADP aggregation; Figure S6. TQ time-dependently increases PS exposure; Figure S7. TQ increases ROS formation, starting from 10 min of incubation; Figure S8. TQ acutely potentiated Trap-6-induced ROS formation; Figure S9. Full blots of Figure 1F.
