# Supplementary material for: Multifaceted Effects of Thymoquinone on Platelet Calcium Homeostasis
Source: Cells. 2025 Nov 20;14(22):1827. doi: 10.3390/cells14221827 (PMC12651197; doi:10.3390/cells14221827)

Supplementary Materials

Figure S1. Structural formulas of TQ, THBQ, thapsigargin, and DTT

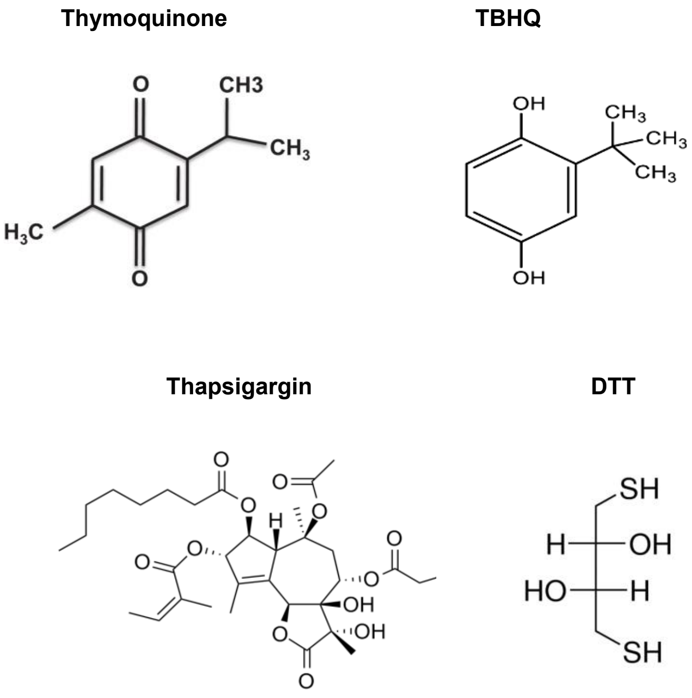

Figure S2. Analysis of platelet aggregation, shape change, and [Ca2+]i mobilization by the Laser Diffraction Method

LaSca-TM laser analyzer registers light scattering intensity (LSI) at the scattering angles from 1º to 12º. In our experiments, we used 1° and 12° due to their high sensitivity for agonist-induced platelet transformations. The platelet shape change was characterized by an increase in the light scatter intensity (LSI) at the scattering angle of 1°. The platelet aggregation was characterized by the LSI increase at the scattering angle of 1° with a simultaneous LSI decrease at the scattering angle of 12°. The area under the curve (AUC) at LSI 12°during three minutes of reaction was used for calculating the aggregation reaction (Fig. S2A), and the velocity of platelet shape change at LSI 1° (V_{shape}) was used for calculating the shape change reaction (Fig. S2B). For elucidation of $[Ca^{2+}]_i$ mobilization (Figure S2C), laser microparticle analyzer LaSca-TM with a 488 nm laser and a fluorescence detector of 527 nm (FL1) was used. PRP was incubated with Fluo-3-AM (10 μM, 60 min, RT) in the dark, diluted in HEPES buffer, and analyzed by fluorescence analysis. Increase in FL1 signal corresponded to $[Ca^{2+}]_i$ rise. For characterization calcium dynamics area under the curve (AUC) was calculated.

Commented [EE1]: Please check that the intended meaning has been retained.

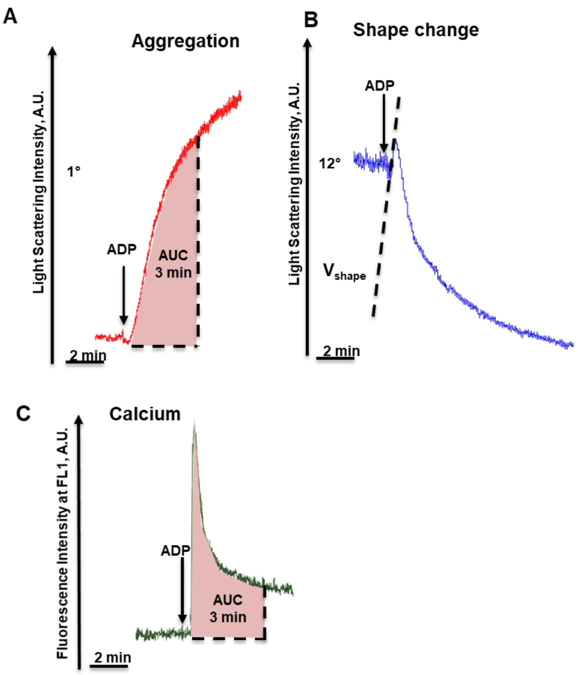

Figure S3. Calculation of $[Ca^{2+}]_i$ concentration by the Laser Diffraction Method

To calibrate $[Ca^{2+}]_i$ concentration in platelets stained by Fluo-3 AM, we used A23187 to equilibrate the extracellular and intracellular calcium concentration. When the Fluo-3 fluorescence intensity (FI) reached maximum (continuous plateau of the FI signal), EGTA (20 mM) was added, and the decrease in FI was registered until it reached the minimum (Figure S3A). Next, using Equation S1 and Fluo-3 K_d 390 nM, the $[Ca^{2+}]_i$ changes were calculated in dynamics (Figure S3B). The initial $[Ca^{2+}]_i$ was estimated to be around 100 nM (Figure S3B), which is in good agreement with the literature. These data confirmed that the fluorescence module of LaSca-TMF can be used for $[Ca^{2+}]_i$ assessment.

(S1) $[Ca^{2+}]_i = Kd(F - F_{min})/(F_{max} - F)$,

where K_d is a dissociation constant for $Ca^{2+,}$ which is reported (according to the manufacturer) to be 390 nM; F_{min} is the fluorescence intensity of the indicator in the absence of calcium; Fmax is the fluorescence of the calcium-saturated indicator; and F is the fluorescence at intermediate calcium levels.

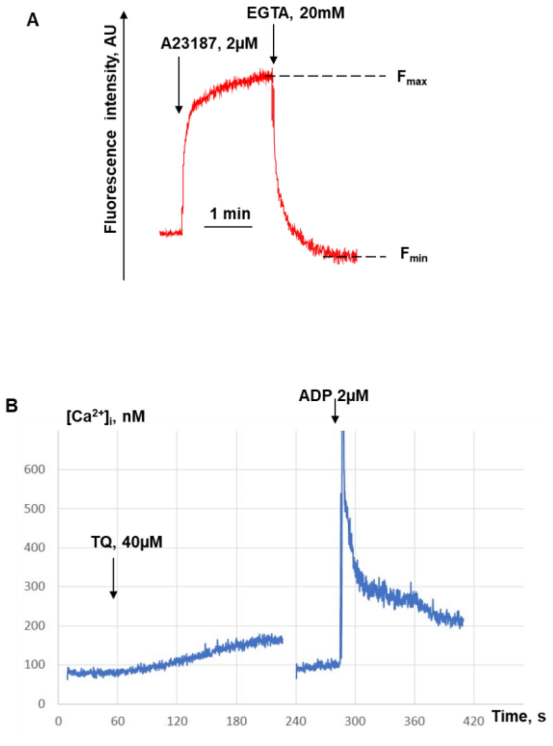

Figure S4. TQ decreases cell esterase activity starting from 30 min of incubation

Platelet viability was analyzed by a marker of the cell esterase activity (Calcein-AM). Washed platelets (2 x 10^7 cell/mL final concentration) were incubated with TQ (40 μM, for the indicated time, at 37 °C), and then C-AM (0.2 μM, 10 min) was added to the cells, and calcein fluorescence was analyzed by flow cytometry.

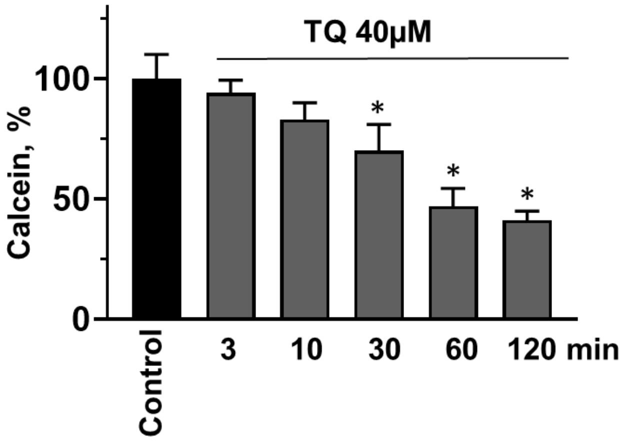

Figure S5. Dithiothreitol reverses TQ-induced inhibition of ADP aggregation

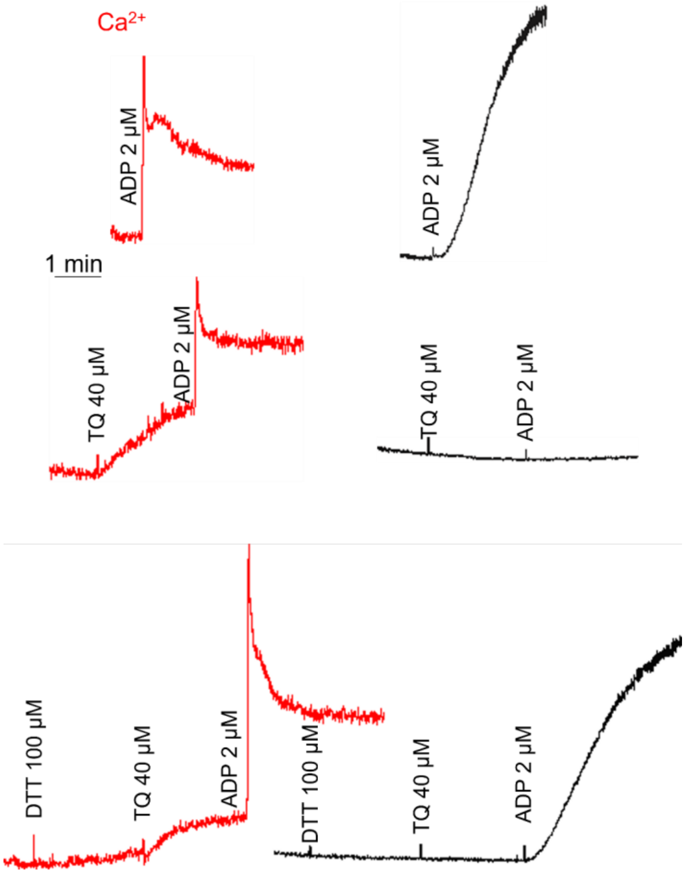

Figure S6. TQ time-dependently increases PS exposure

Phosphatidylserine exposure (PS) was measured by Annexin-V-PE binding. Platelets were incubated with Annexin-V-PE (1:10) and then TQ (40 μM, for the indicated time, at 37 °C) was added to the cells. Annexin-V fluorescence was analyzed by flow cytometry.

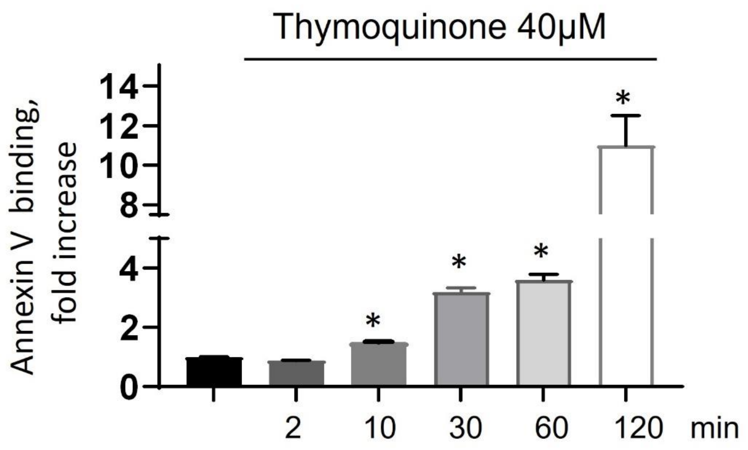

Figure S7. TQ increases ROS formation, starting from 10 min of incubation

Formation of reactive oxygen species (ROS) in platelets was analyzed by DCF-DA. Washed platelets (2 x 10^7 cell/mL final concentration) were incubated with DCF-DA (10 µM, 30 min, at 37 °C), then TQ (40 µM, for the indicated time) was added to the cells. DCF-DA fluorescence was analyzed by flow cytometry. Data (means ±SD, n = 6) are presented as fold changes, where the control is taken as 1.

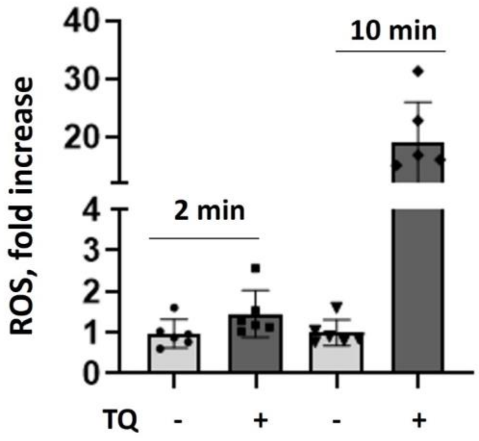

Figure S8. TQ acutely potentiated Trap-6-induced ROS formation

Formation of reactive oxygen species (ROS) in platelets was analyzed by DCF-DA. Washed platelets (2 x 10^7 cell/mL final concentration) were incubated with DCF-DA (10 μM, 30 min, at 37 °C). Platelets were incubated with TQ (40 μM, 2 min), stimulated with Trap-6 (10 μM, 2 min), or preincubated with TQ (40 μM, 2 min) and then stimulated with Trap-6 (10 μM, 2 min). DCF-DA fluorescence was analyzed by flow cytometry. Data (means ±SD, n = 5, * significant differences from control, + significant differences from Trap-6 alone) are presented as fold changes, where the control is taken as 1.

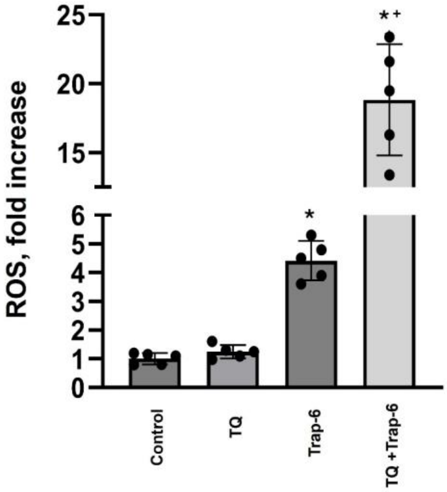

Figure S9. Full blots of Figure 1F.

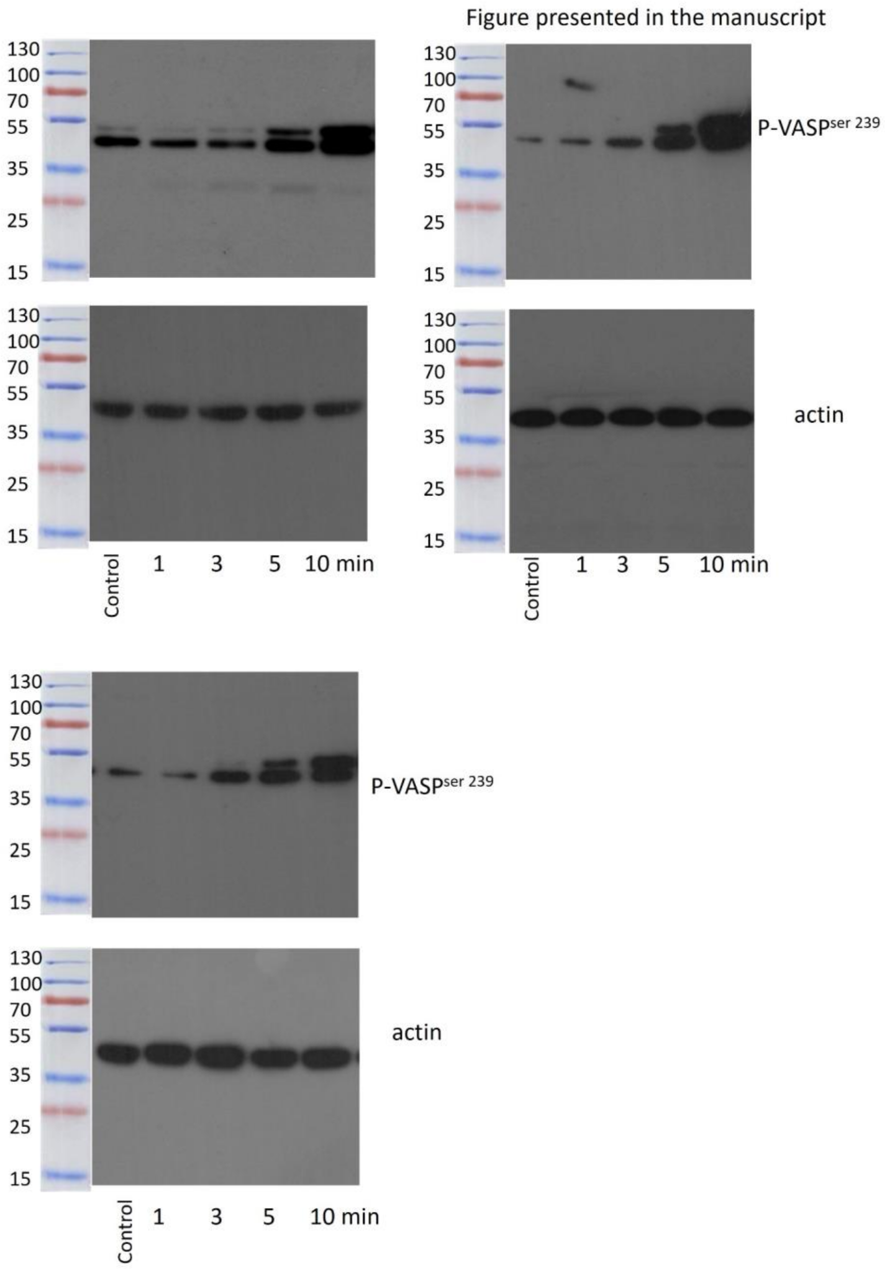

Supplement: Supplementary file 1 [file cells-14-01827-s001.zip › cells-3976237-supplementary.pdf]
